# Impact of Stress Hyperglycemia on No-Reflow Phenomenon in Patients with ST Elevation Myocardial Infarction Undergoing Primary Percutaneous Coronary Intervention

**DOI:** 10.5334/gh.1111

**Published:** 2022-03-29

**Authors:** Mohamed Khalfallah, Dina A. Maria, Amany Allaithy

**Affiliations:** 1 Cardiovascular department, Tanta University, EG

**Keywords:** Impact, stress hyperglycemia, no-reflow phenomenon, ST elevation myocardial infarction, primary percutaneous coronary intervention

## Abstract

**Background:**

Stress hyperglycemia is a common finding during acute myocardial infarction and associated with poor prognosis. To reduce the occurrence of no-reflow, prognostic factors must be identified before primary percutaneous coronary intervention (PPCI). Our objective was to investigate the impact of stress hyperglycemia in non-diabetic and diabetic patients on no-reflow phenomenon after PPCI.

**Methods:**

The study comprised 480 patients with ST elevation myocardial infarction (STEMI) who were managed by PPCI. Patients were classified into two groups according to thrombolysis in myocardial infarction (TIMI) flow grade: Group I (Patients with normal flow, TIMI 3 flow) and Group II (Patients with no-reflow, TIMI 0-2 flow). Patients were analyzed for clinical outcomes including mortality and major adverse cardiac events.

**Results:**

Incidence of stress hyperglycemia was 14.8% in non-diabetic patients and 22.2% in diabetic patients; the incidence of no-reflow phenomenon was 13.5% and no-reflow was significantly higher in patients with stress hyperglycemia. Multivariate regression analysis identified the independent predictors of no-reflow phenomenon: stress hyperglycemia OR 3.247 (CI95% 1.656–6.368, P = 0.001), Killip class >1 OR 1.893 (CI95% 1.004–3.570, P = 0.049) and cardiogenic shock OR 3.778 (CI95% 1.458–9.790, P = 0.006).

**Conclusion:**

Stress hyperglycemia was associated with higher incidence of no-reflow phenomenon. The independent predictors of no-reflow were stress hyperglycemia, Killip class >1 and cardiogenic shock.

## Introduction

Stress hyperglycemia is a frequent complication in the setting of acute myocardial infarction and affects patients with and without established diabetes mellitus (DM). Stress hyperglycemia is temporarily increasing in blood glucose levels during critical illness [1]. Stress hyperglycemia is a common finding in patients with ST elevation myocardial infarction (STEMI), and has been described to occur in up to 50% of many hospital units [2]. Stress hyperglycemia is a predictor of in-hospital and long-term adverse outcomes in patients with STEMI, irrespective of diabetic status of those patients [3]. The increased mortality in patients with stress hyperglycemia might be explained by a larger infarct size, a high incidence of congestive heart failure, and cardiogenic shock [4]. Moreover, electrophysiological alterations with significant QT elongation may favor the occurrence of arrhythmias, whose outcome could be fatal [5].

Hyperglycemia occurring at the onset of acute myocardial infarction seems to be related to the stress mechanism, which is recognized by high free fatty acids, steroid hormones and insulin resistance [6]. Stress hyperglycemia seems to be a protective mechanism by creating a new glucose balance, allowing a higher blood glucose diffusion gradient that increases cellular glucose uptake in the face of mal-distributed micro-vascular flow [7]. Actually, this mechanism becomes injurious to the mitochondria, increases oxidative stress with more damage to the cells. Furthermore, elevated blood glucose also could be a marker of existing insulin resistance and/or beta-cell failure. This may contribute to poor prognosis as it causes alterations in blood coagulation with more likely to cause thrombosis. Moreover, it worsens endothelial function with amplification of inflammatory immune reactions and worse functional cardiac outcome. Stress hyperglycemia is independently associated with impaired left ventricular function with a larger infarct size due to an increased incidence of the no-reflow phenomenon [8].

No-reflow phenomenon, the major adverse complication of primary percutaneous coronary intervention (PPCI), causes poor prognosis with greater morbidity and mortality due to poor healing of the infarction, adverse left ventricular remodeling with more occurrence of congestive heart failure [9]. At present, the exact mechanism of no-reflow phenomenon remains unclear, but some clinical and laboratory findings suggesting that, it is related to the embolism of the capillary bed, endothelial dysfunction, ischemic injury, oxygen free radical production, inflammatory reaction, calcium overload, stress response and other factors. Although the reperfusion techniques for STEMI are continually improving, no-reflow can still lead to poor prognosis [10]. Therefore, to illustrate the independent predictors of no-reflow phenomenon before PPCI in patients with STEMI is critically useful to provide guidance for interventionists to prevent the occurrence of no-reflow. So, the objective of the present study was to investigate the impact of stress hyperglycemia in non-diabetic and diabetic patients on no-reflow phenomenon after PPCI and to assess the independent predictors of no-reflow phenomenon.

## Patients and methods

### Study population

This study was a prospective study with 480 patients recruited to the study at initial presentation with STEMI who were managed by PPCI and received the standard care in our cardiovascular department. Patients were classified into two groups according to the occurrence of no-reflow phenomenon: Group I (Patients with TIMI 3 flow) and Group II (Patients with no-reflow phenomenon, TIMI flow ≤2). All patients included in the study signed a written informed consent and a code number was given for every patient pointed to his address and telephone number. The study was approved by local research ethics committee of faculty of medicine, Tanta University, and was in agreement with the principles of Declaration of Helsinki II. The diagnosis of STEMI was established according to the 4th universal definition of myocardial infarction as: typical rise of biochemical markers of myocardial necrosis with at least one of the following: (i) ischemic symptoms as chest discomfort or pain >20 min within less than 24 hours. (ii) ECG changes indicative of ischemia: new ST elevation at the J-point in two contiguous leads. (iii) New onset LBBB. (iv) Imaging evidence of new loss of viable myocardium or new regional wall motion abnormality [11].

#### Inclusion criteria

Patients presented to our cardiovascular department with STEMI within the first 24 hours who were suitable for revascularization by PPCI either non-diabetics or diabetics with controlled diabetes mellitus (HbA1c < 6.5%).

#### Exclusion criteria

Patients presented with high HbA1c level (HbA1c > 6.5%) were excluded from the study to exclude patients with uncontrolled DM. Moreover, patients with new onset DM, their blood glucose levels still rising after the period of stress were also excluded from the study. Patients with missed myocardial infarction with the onset of symptoms more than 24 hours were excluded also, as this increases the risk of no-reflow that may influence our result. Moreover, patients with previous coronary artery bypass graft surgery or indicated for it. We excluded also patients with severe hepatic or renal impairment, active infection, mental or intellectual impairment and patients who were taking glucocorticoid therapy at the time of admission.

### Demographic, clinical and laboratory data

All patients were subjected to full history taking about atherosclerosis risk factors e.g., hypertension, dyslipidemia, obesity and smoking. History of comorbidities was interrogated e.g., chronic kidney disease and previous myocardial infarction. The total ischemia time was calculated. Full physical examination of the patients was performed and resting standard twelve lead ECG was done for all patients. Echocardiographic examination was done after the procedure for all patients with Vivid E9 dimension (General Electric Medical Systems, Horten, Norway) with assessment of left ventricular ejection fraction by Simpson’s method. Venous blood samples were collected and used for laboratory investigations including; random blood glucose level, HbA1c%, complete lipid profile, serum creatinine level before and after the procedure, hemoglobin level and CK-MB. DM was defined as HbA1c ≥ 6.5%, fasting plasma glucose ≥126 mg/dl or 2 h plasma glucose ≥200 mg/dl [12]. Patients without previously known history of DM but with HbA1c ≥6.5% on admission was classified as having newly detected DM and were excluded from the study [12]. Stress hyperglycemia was defined according to the American diabetes association guidelines that described stress hyperglycemia as having a random glucose level greater than 140 mg/dl at any given time in hospitalized patients who were not known to have DM [12]. According to the definition used in previous studies of stress hyperglycemia in diabetic patients [2,13,14], stress hyperglycemia in diabetics was defined as a blood glucose level at admission >198 mg/dl.

#### Coronary angiography

On admission, patients received aspirin tablets 300 mg, clopidogrel 600 mg or ticagrelor 180 mg and intravenous unfractionated heparin. PPCI was done by either radial or femoral approach according to operator preference. Standard left and right coronary angiograms were obtained. Two experienced interventionists assessed the diagnostic coronary angiography. After identification of the anatomy, culprit vessel, pre-procedural TIMI flow and thrombus burden, revascularization of the culprit vessel was done. Angiographic coronary thrombus burden was classified using TIMI thrombus grades as follows: Grade 0: no thrombus, Grade 1: possible thrombus, Grade 2: the thrombus’ greatest dimension is <1/2 vessel diameter, Grade 3: greatest dimension >1/2 to <2 vessel diameters, Grade 4: greatest dimension >2 vessel diameters, Grade 5: total vessel occlusion due to thrombus [15]. The patients were stratified into low thrombus burden (Grades ≤ 2), moderate thrombus burden (Grades = 3) and high thrombus burden groups (Grades ≥ 4) according to final thrombus score. Aspiration catheter and glycoprotein IIb/IIIa inhibitors were used according to operator judgment. TIMI flow post-procedural was reported. The contrast media used was non-ionic low-osmolar and the volume of contrast agent was measured for each patient.

#### Endpoints

The Primary end point of this study was the occurrence of no-reflow phenomenon which has various definitions. Classically, it is considered to be the lack of myocardial perfusion despite opening up the epicardial coronary vessel in the setting of PPCI with TIMI flow in the artery ≤ 2, despite the successful dilatation and the absence of mechanical complications such as dissection, spasm or evident distal embolization seen angiographically after completing of the procedure [16]. The secondary endpoints were the occurrence of mortality or major adverse cardiac events in the form of heart failure, major bleeding, cardiogenic shock, cardiac arrest, re-infarction and contrast- induced nephropathy. The patients were followed up for three months.

#### Statistical analysis

Statistical analysis was done using SPSS 23, IBM, Armonk, NY, United States of America. Quantitative variables were expressed as mean± standard deviation. Qualitative data were expressed as frequency and percentage. Student’s t test was used to assess the significance between the two groups in quantitative data. Chi-square (X^2^) test was used to assess the significance between two qualitative parameters. P value <0.05 was considered statistically significant. Multivariate regression analysis was performed to detect the independent predictors of no-reflow phenomenon. Pearson’s correlation analysis was performed to test the correlation between random blood glucose and TIMI flow grade. Receiver Operating Characteristic curve (ROC-curve) analysis was done to detect the best cut-off values of random blood sugar in non-diabetic and diabetic patients for the prognostic impact of no-reflow phenomenon. Kaplan-Meier survival analysis was done for comparing survival function in the two groups.

## Results

The present study included 480 patients with STEMI admitted to our cardiovascular department and subjected to PPCI. Patients were divided into two groups according to the occurrence of no-reflow phenomenon: Group (I) 415 patients (86.5%) with TIMI 3 flow and Group (II) 65 patients (13.5%) with no-reflow phenomenon, TIMI flow ≤2. Patients with no-reflow phenomenon were older in age than group I with (P value = 0.037). Systolic blood pressure was significantly lower in no-reflow group with (P value = 0.001). The number of patients with atrial fibrillation and patients with Killip class >1 was significantly higher in no-reflow group with (P value = 0.049, 0.035 respectively). The number of patients with stress hyperglycemia in non-diabetic and diabetic patients was significantly higher in no-reflow group with (P value = 0.001, 0.008 respectively). As regarding laboratory results random blood sugar was significantly higher in non-diabetic and diabetic patients in no-reflow group with (P value = 0.001, 0.003 respectively). Concerning clinical outcome mortality was higher in Group II with (P value = 0.040). Cardiogenic shock, cardiac arrest and contrast-induced nephropathy were more predominant in Group II with (P value = 0.001, 0.014, 0.048 respectively) with no other statistically significant difference between both groups regarding other demographic, basal clinical characteristics, laboratory results and clinical outcome as shown in ***Tables 1, 2*** and ***5***.

**Table 1:** Demographic and baseline clinical characteristics of all patients in the two groups.


	GROUP I (TIMI 3 FLOW) (N = 415) (86.5%)	GROUP II (NO-REFLOW) (N = 65) (13.5%)	P VALUE

Age, years	54.99 ± 9.06	57.52 ± 9.40	0.037*

Male gender, n (%)	212 (51.1%)	35 (53.8%)	0.679

Hypertension, n (%)	154 (37.1%)	25 (38.5%)	0.834

Diabetes mellitus, n (%)	115 (27.7%)	20 (30.8%)	0.610

Smoking, n (%)	112 (27.0%)	24 (36.9%)	0.098

Dyslipidemia, n (%)	154 (37.1%)	26 (40.0%)	0.654

Prior myocardial infarction, n (%)	32 (7.7%)	4 (6.2%)	0.658

Chronic kidney disease, n (%)	51 (12.3%)	11 (16.9%)	0.300

BMI, (kg/m^2^)	25.03 ± 3.22	25.37 ± 3.08	0.435

Systolic BP, mmHg	119.6 ± 19.5	108.5 ± 24.3	0.001*

Diastolic BP, mmHg	77.45 ± 12.7	74.26 ± 13.4	0.064

Heart rate, (bpm)	72.24 ± 13.3	74.78 ± 13.9	0.156

Atrial fibrillation, n (%)	47 (11.3%)	13 (20.0%)	0.049*

Killip class > 1	75 (18.1%)	19 (29.2%)	0.035*

LVEF, (%)	45.36 ± 4.92	45.12 ± 5.75	0.725

Non-diabetic patients with SH, n (%)	34 (11.3%)	17 (37.8%)	0.001*

Non-diabetic patients with euglycemia, n (%)	266 (88.7%)	28 (62.2%)	

Diabetic patients with SH, n (%)	21 (18.3%)	9 (45.0%)	0.008*

Diabetic patients with euglycemia, n (%)	94 (81.7%)	11 (55.0%)	


BMI: body mass index; BP: blood pressure; LVEF: left ventricular ejection fraction; SH: stress hyperglycemia; *: significant P value.

**Table 2:** Laboratory results of all patients in the two groups.


	GROUP I (TIMI 3 FLOW) (N = 415) (86.5%)	GROUP II (NO-REFLOW) (N = 65) (13.5%)	P VALUE

RBS in non-diabetic patients with euglycemia, mg/dl	114.9 ± 16.5	118.0 ± 20.2	0.348

RBS in non-diabetic patients with SH, mg/dl	174.7 ± 23.9	201.4 ± 29.6	0.001*

RBS in diabetic patients with euglycemia, mg/dl	168.8 ± 22.4	178.7 ± 16.7	0.163

RBS in diabetic patients with SH, mg/dl	246.9 ± 50.4	302.2 ± 8.33	0.003*

Hemoglobin level, g/dL	12.32 ± 1.34	12.09 ± 1.42	0.221

Creatinine pre-procedure, mg/dl	1.029 ± 0.24	1.055 ± 0.28	0.427

Creatinine post-procedure, mg/dl	1.190 ± 0.47	1.220 ± 0.49	0.633

CK-MB, U/L	89.25 ± 43.4	85.08 ± 43.5	0.472

HbA1c in non-diabetic patients with euglycemia, %	5.18 ± 0.390	5.13 ± 0.394	0.577

HbA1c in non-diabetic patients with SH, %	5.16 ± 0.452	5.20 ± 0.466	0.764

HbA1c in diabetic patients with euglycemia, %	6.35 ± 0.161	6.35 ± 0.129	0.929

HbA1c in diabetic patients with SH, %	6.40 ± 0.122	6.44 ± 0.072	0.433

Total cholesterol, mg/dl	219.9 ± 54.4	229.4 ± 56.9	0.194

HDL, mg/dl	39.13 ± 8.18	39.84 ± 8.88	0.517

LDL, mg/dl	131.9 ± 24.8	130.5 ± 22.0	0.660

Triglycerides, mg/dl	180.8 ± 39.5	182.8 ± 37.4	0.697


RBS: random blood sugar; SH: stress hyperglycemia; CK-MB: Creatine kinase myocardial band; HDL: high density lipoprotein; LDL: low-density lipoprotein; *: significant P value.

Concerning angiographic results, there was no statistically significant difference between both groups regarding the total ischemia time, initial TIMI flow, the culprit vessel, the length of the lesion, thrombus burden and the volume of contrast agent used during the procedure. However, there was statistically significant difference regarding post-procedural TIMI flow, the number of patients in no-reflow group with TIMI flow = 0 was 22 patients (33.8%), TIMI flow = 1 was 28 patients (43.1%) and TIMI flow = 2 was 15 patients (23.1%). Moreover, there was statistically significant difference between both groups regarding the need for aspiration catheters, which was more in Group II in addition to glycoprotein IIb/IIIa inhibitors with (P value = 0.001, 0.001 respectively) as shown in ***Table 3***.

**Table 3:** Angiographic results of all patients in the two groups.


	GROUP I (TIMI 3 FLOW) (N = 415) (86.5%)	GROUP II (NO-REFLOW) (N = 65) (13.5%)	P VALUE

The total ischemia time, h	5.20 ± 2.94	5.11 ± 2.86	0.813

Initial TIMI flow			

0	331 (79.8%)	53 (81.5%)	0.956

1	34 (8.2%)	4 (6.2%)	

2	19 (4.6%)	3 (4.6%)	

3	31 (7.5%)	5 (7.7%)	

Thrombus burden			

Low	141 (34.0%)	23 (35.4%)	0.748

Moderate	160 (38.6%)	22 (33.8%)	

High	114 (27.5%)	20 (30.8%)	

Aspiration catheter	31 (7.5%)	13 (20%)	0.001*

Glycoprotein IIb/IIIa inhibitors	47 (11.3%)	55 (84.6%)	0.001*

Reperfusion type			

Balloon angioplasty	24 (5.8%)	35 (53.8%)	0.001*

Direct stenting	116 (28.0%)	12 (18.5%)	

Stenting after pre-dilatation	275 (66.3%)	18 (27.7%)	

Length of the lesion, mm	20.68 ± 5.11	21.29 ± 6.15	0.385

Volume of contrast agent, (ml)	182.5 ± 65.1	170.3 ± 76.3	0.170

Culprit vessel			

LM coronary artery, n (%)	3 (0.7%)	2 (3.1%)	0.082

LAD coronary artery, n (%)	168 (40.5%)	24 (36.9%)	0.586

CX coronary artery, n (%)	124 (29.9%)	23 (35.4%)	0.371

Right coronary artery, n (%)	120 (28.9%)	16 (24.6%)	0.474

Post-procedural TIMI flow			

0	0 (0.0%)	22 (33.8%)	0.001*

1	0 (0.0%)	28 (43.1%)	

2	0 (0.0%)	15 (23.1%)	

3	415 (100%)	0 (0.0%)	


TIMI: thrombolysis in myocardial infarction; LM: left main; LAD: left anterior descending; CX: circumflex; *: significant P value.

**Figure 1:**
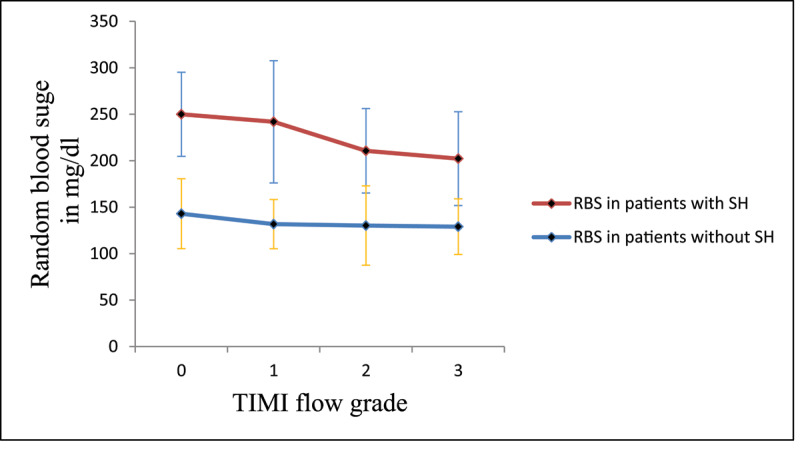
Relationship between random blood sugar in patients with and without stress hyperglycemia and TIMI flow grade.

**Figure 2:**
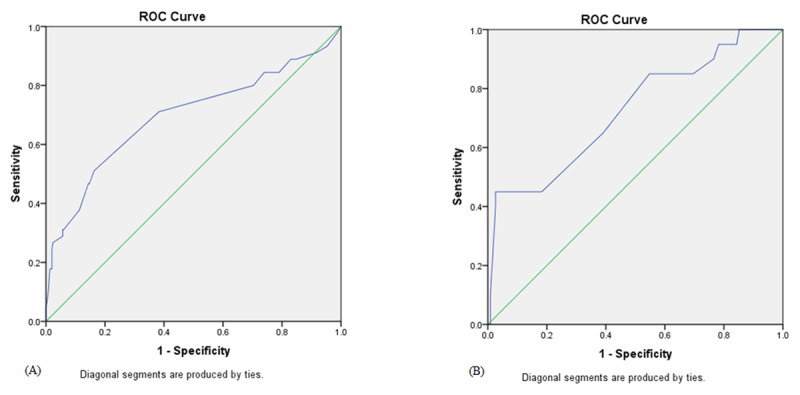
ROC curve analysis for random blood sugar in non-diabetic **(A)** and diabetic patients **(B)** for prediction of no-reflow phenomenon after primary percutaneous coronary intervention.

**Figure 3:**
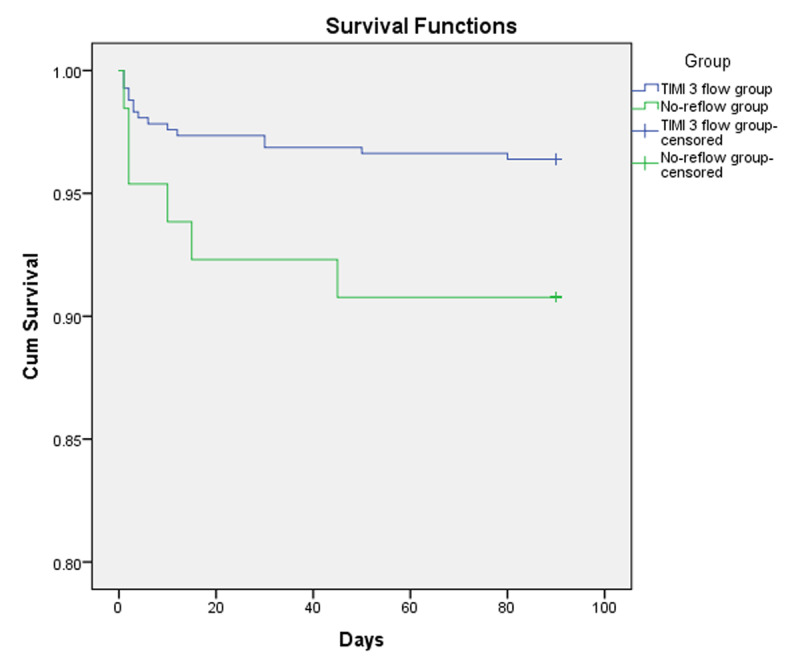
Kaplan-Meier curve showing survival functions of the patients in Group I (TIMI 3 flow) and Group II (no-reflow group).

Multivariate regression analysis was performed to identify the independent predictors of no-reflow phenomenon with the following results: stress hyperglycemia OR 3.247 (CI95% 1.656–6.368, P = 0.001), Killip class >1 OR 1.893 (CI95% 1.004–3.570, P = 0.049) and cardiogenic shock OR 3.778 (CI95% 1.458–9.790, P = 0.006) were the independent predictors of no-reflow phenomenon as shown in ***Table 4***. Pearson’s correlation analysis was performed to test the correlation between random blood glucose and TIMI flow grade and showed a significant negative correlation between them (r = –271 and P value = 0.001) as shown in ***Figure 1***. The ROC analysis provided a cut-off value for random blood sugar >160 mg/dl in non-diabetic patients to predict the no-reflow phenomenon with sensitivity = 51.1%, specificity = 83.7%, positive predictive value = 31.9% and negative predictive value = 91.9%. The ROC analysis provided a cut-off value for random blood sugar >240 mg/dl in diabetic patients to predict no-reflow phenomenon with sensitivity = 45%, specificity = 97.4%, positive predictive value = 75% and negative predictive value = 91.1% as shown in ***Figure 2***. Kaplan-Meier survival analysis was done showing better survival function in Group I as shown in ***Figure 3***.

**Table 4:** Multivariate regression analysis for the independent predictors of no-reflow phenomenon.


	MULTIVARIATE ANALYSIS		P VALUE

	OR	(95% CI)	

Age > 60 years	1.019	0.550–1.886	0.953

Stress hyperglycemia	3.247	1.656–6.368	0.001*

Killip class >1	1.893	1.004–3.570	0.049*

Atrial fibrillation	1.161	0.504–2.677	0.726

Cardiogenic shock	3.778	1.458–9.790	0.006*

Cardiac arrest	2.595	0.758–8.876	0.129


**Table 5:** Clinical outcomes of all patients in the two groups.


	GROUP I (TIMI 3 FLOW) (N = 415) (86.5%)	GROUP II (NO-REFLOW) (N = 65) (13.5%)	P VALUE

**Mortality, n (%)**	15 (3.6%)	6 (9.2%)	0.040*

**Major bleeding, n (%)**	5 (1.2%)	1 (1.5%)	0.822

**Cardiogenic shock, n (%)**	14 (3.4%)	12 (18.5%)	0.001*

**Cardiac arrest, n (%)**	9 (2.2%)	5 (7.5%)	0.014*

**Heart failure, n (%)**	48 (11.6%)	13 (20.0%)	0.058

**Contrast-induced nephropathy, n (%)**	42 (10.1%)	12 (18.5%)	0.048*

**Re-infarction, n (%)**	11 (2.7%)	4 (6.2%)	0.131


## Discussion

Stress hyperglycemia in patients with acute myocardial infarction seems to be transition phenomenon of detrimental effects induced by the acute release of catecholamine, cytokines, and cortisol in the acute stage of myocardial infarction rather than a reflection of underlying gluco-metabolic state of the patient [17]. Hyperglycemia can occur because of increasing levels of stress hormones e.g., catecholamines, steroids, glucagon and decreasing levels of insulin due to stress. Moreover, increasing levels of catecholamines can inhibit pancreatic beta cells to secrete insulin [18]. This excited autonomic nervous system can lead to heart failure, hemodynamic instability and larger infarct size. The acute increase of plasma glucose level causes several adverse effects, including endothelial dysfunction, oxidative stress, inflammation, hypercoagulability state and apoptosis that may contribute to worse outcomes in patients with acute myocardial infarction [17,18,19].

The incidence of stress hyperglycemia in the current study was 14.8% in non-diabetic patients and 22.2% in diabetic patients. In agreement to our results Nakamura et al. [20], reported that glucose level > 198 mg/dl (11.0 mmol/L) on admission was observed in 31% of all patients and 15% of non-preexisting diabetic patients when studying the impact of acute hyperglycemia during primary stent implantation in patients with acute myocardial infarction. Deckers and coworkers analyzed a large number of patients (11,324), of whom 41% had elevated blood glucose level at admission > 140 mg/dl (7.8 mmol/L) [21]. The incidence of no-reflow phenomenon in the current study was 13.5% and was significantly higher in patients with stress hyperglycemia. Higher blood glucose levels on admission were associated with reduced TIMI flow in patients with STEMI after PPCI. The no-reflow phenomenon is characterized by impairment of myocardial perfusion despite reopening of the epicardial coronary artery. The relationship between no-reflow phenomenon and stress hyperglycemia can be explained by a lot of mechanisms. First, hyperglycemia increases the level of intercellular adhesion molecule-1 (ICAM-1) and P-selectin that increases the adhesion of leukocytes to capillaries with increasing the obstruction of the capillary bed [22]. Moreover, hyperglycemia increases the occurrence of micro-thrombi, one of the key reasons of no-reflow phenomenon. Micro-emboli during PPCI procedure may be a major cause of micro-vascular dysfunction. Micro-vascular plugging by platelets and neutrophils due to high platelet activity and much thrombus burden, reperfusion injury, ischemic injury, endothelial dysfunction, inflammation, oxidative stress, interstitial edema and swelling of myocardial cells compressing microvascular vessels may be involved in its pathogenesis and could be enhanced by hyperglycemia [23,24]. The possible role of hyperglycemia in the activation of blood coagulation has been studied previously. It emerges that acute glycemic variations are matched with alterations in coagulation cascade that are likely to cause thrombosis. Stress hyperglycemia induces a shortening of the fibrinogen half-life, and increases in fibrinopeptide A, fragments of pro-thrombin, in factor VII, and in platelet aggregation. All phenomena suggesting increased activation of thrombosis [25,26,27,28,29,30].

As regarding the mean age of patients in the current study, it was significantly higher in no-reflow group. Patients with advanced age have a tendency to be associated with diffuse atherosclerosis, more coronary calcification, distal microembolization and increased comorbidities. Patients with atrial fibrillation were significantly higher in no-reflow group that may lead to hemodynamic compromise and impairment of coronary flow [31]. In the current study we noticed that mortality was higher in no-reflow group and with further analysis we found that mortality was higher in non-diabetic patients with stress hyperglycemia than diabetic patients with stress hyperglycemia. Several studies have validated that hyperglycemia in the setting of STEMI is an independent predictor of mortality regardless of diabetic status [2,32,33]. In agreement to our results two of these studies revealed that non-diabetic patients with stress hyperglycemia have higher mortality than diabetic patients [2,33]. This may be clarified by less aggressive medical treatment in the non-diabetic cohort, this justifies the importance of proper identification and management of stress hyperglycemia in non-diabetic patients with STEMI. In HORIZONS-AMI trial [34], hyperglycemia was defined as serum glucose level more than 156 mg/dl and was associated with higher mortality rates and higher incidence of re-infarction and bleeding after PPCI. Kosiborod et al. [33], who studied the relation between admission glucose and mortality in elderly patients with and without recognized diabetes hospitalized with acute myocardial infarction reported that, non-diabetic patients who had admission blood glucose levels > 200 mg/dl, had mortality rate similar to that of patients who had established diabetes mellitus (42.6% and 43.1% respectively). But in contrast to our study Kim et al. [35], found that diabetics have significantly higher in-hospital mortality rate in comparison to non-diabetics.

The total ischemia time (the time from chest pain onset to balloon dilatation) reflects the degree of myocardial injury and necrosis. Therefore, the longer total ischemia time can cause swelling of distal capillary endothelia, neutrophil occlusion with more severe damage to the microcirculation and higher the likelihood of no-reflow. Reffelmann et al., stated that changes in ultrastructure of myocardial capillary endothelia were directly related to the occurrence of no-reflow phenomenon. Generally, the pathological process of myocardial cell necrosis in the infarcted area of the myocardium was basically completed after 6 hours of coronary artery occlusion. The longer the time of vascular occlusion, the worse the reperfusion [36,37,38]. However, the results of the present study showed that the total ischemia time was equal in both groups and excluded the total ischemia time to be a risk factor for no-reflow in this study. Numerous studies have reported that high thrombus burden in the culprit vessel is an important risk factor of no-reflow [39,40]. Yip et al. [41], who studied the angiographic morphologic features of infarct-related arteries in 794 patients with STEMI who underwent PPCI showed that high thrombus burden was an independent predictor of no-reflow. However, in the current study the thrombus burden showed no statistically significant difference between both groups and was excluded to be a risk factor for no-reflow. As regarding reperfusion type, it was a matter of operator experience to judge using direct stenting, stenting after pre-dilatation or balloon angioplasty only according to the circumstances of every case e.g. Initial TIMI flow, diameter of the culprit vessel, thrombus burden, presence of plaque or not and TIMI flow grade after pre-dilatation.

Contrast-induced nephropathy in the current study was higher in patients with no-reflow and significantly higher in patients with stress hyperglycemia. The previous studies stated that fluctuations in blood glucose levels more harmful than chronically elevated glucose levels [42,43,44], these fluctuations can increase apoptosis and oxidative stress. So, stress hyperglycemia may aggravate the negative effects of contrast media exposure and increases the risk of contrast-induced nephropathy. Although contrast-induced nephropathy could be seen with the use of higher doses of contrast media [45,46], in the current study the volume of contrast agent showed no statistically significant difference between both groups. In agreement to our results Marenzi et al. [47], who studied the effect of acute hyperglycemia and its relation to contrast-induced nephropathy in patients with STEMI after PPCI stated that, patients with acute hyperglycemia had a 2-fold higher incidence of contrast-induced nephropathy than those without acute hyperglycemia [34].

Killip class >1 suggests that evidence of heart failure has been found. The correlation between heart failure and no-reflow phenomenon is a highly complex mechanism involving neurohumoral activation that subsequently leads to imbalance between nitric oxide and reactive oxygen species. Abundant formation of reactive oxygen species and reduced bioavailability of nitric oxide within the vascular wall can play an important role in endothelial dysfunction which is the basic mechanism of pre-existing microvascular dysfunction [48].

## Conclusion

Stress hyperglycemia is a strong predictor of morbidity and mortality in patients with STEMI who were managed by PPCI. Stress hyperglycemia is associated with a higher incidence of no reflow phenomenon. In the current study the independent predictors of no-reflow phenomenon were stress hyperglycemia, Killip class >1 and cardiogenic shock. The cut-off value for random blood sugar to predict the no-reflow phenomenon in non-diabetic patients was >160 mg/dl and >240 mg/dl in diabetic patients. Blood glucose levels should be monitored closely in patients with STEMI regardless of diabetic status of the patient.
